# Sodium and Potassium Mixed Effects on the Viscoelastic Behavior of Silicate Glasses

**DOI:** 10.3390/ma18061337

**Published:** 2025-03-18

**Authors:** Fucheng Wu, Yonggang Huang, Haizheng Tao, Peng Jiao, Ziyang Xiao, Jinsheng Jia

**Affiliations:** 1 Institute of Special Glass Fiber & Optoelectronic Functional Materials, China Building Materials Academy, Beijing 100024, China; 281509@whut.edu.cn (F.W.); huangyonggang@cbma.com.cn (Y.H.);; 2 State Key Laboratory of Silicate Materials for Architectures, Wuhan University of Technology, Wuhan 430070, China; 3 Key Laboratory of Special Optoelectronic Materials for Building Materials Industry, China Building Materials Academy, Beijing 100024, China

**Keywords:** optical fiber imaging array, optical fiber clad glass, viscoelasticity, mixed alkali effect of Na^+^ and K^+^

## Abstract

This study investigated the viscoelastic behavior and structural evolution of silicate glasses with the molar composition 70SiO_2_·(30 − x) Na_2_O·xK_2_O, where the molar ratio *r* = *x*/30 varied between 0, 0.25, 0.5, and 0.75. A notable “V”-shaped trend in relaxation activation energy (Δ*H*_Gt_) was observed, with the energy reaching a minimum of 163.14 kJ/mol at *r* = 0.5. This trend exhibited a synergistic mixed alkali effect that significantly affected the viscoelastic properties of the glass. Raman spectroscopy analysis revealed dynamic structural reorganization within the [SiO_4_] network, transitioning from Q^4^ to Q^3^ for *r* < 0.5 and reverting to Q^4^ for *r* > 0.5 as the K_2_O content increased. These structural transformations provide atomic-scale evidence for the observed viscoelastic behavior. The findings offer critical insights into the mixed alkali effect on viscoelasticity, establishing a theoretical foundation for optimizing clad materials in optical fiber imaging arrays.

## 1. Introduction

Viscoelasticity refers to the deformation behavior of materials that exhibit both viscosity and elasticity under external forces. The viscoelastic behavior of glass during the thermal forming process directly affects the forming precision and optical performance of high-precision glass components [1,2]. Optical fiber imaging arrays are two-dimensional imaging materials fabricated from three distinct glasses (optical fiber core glass, optical fiber clad glass, and light-absorbing glass) via thermoforming processes—including stretching, fusion pressing, and twisting—at the forming temperature. During high-temperature forming, the viscoelasticity of these three glasses and the compatibility between their viscoelastic properties critically affect the imaging quality, manifesting as geometric distortions and dark spots, which significantly reduce the product qualification rate and result in substantial economic losses [3].

From a microscopic perspective, an optical fiber imaging array consists of millions of glass fibers with diameters ranging from 4 to 10 μm, with each fiber serving as an individual imaging unit. In recent years, with the increasing demand for high-resolution and high-contrast imaging in advanced optoelectronic detection equipment, the performance requirements for optical fiber imaging arrays have continuously increased. However, internal defects in the arrays remain a significant factor limiting the imaging quality for detection and identification [4,5,6,7,8]. The optical fiber clad glass, which is composed of a multi-component silicate glass, can confine light within the fiber core through total internal reflection, ensuring that light signals do not leak during transmission [9]. During high-temperature forming, the clad glass undergoes deformation, including elongation, void filling, and twisting adjustments, influenced by the stretching, pressing, and twisting forces. These deformations must match with the core glass to ensure array formation and maintain total internal reflection, while also significantly affecting the distribution and extent of internal defects in the array.

The deformation of multicomponent silicate glass is inherently time- and stress-dependent, exhibiting nonlinear coupling between elastic recovery and viscous flow. After the external force is removed, the deformation cannot fully recover, and so the relationship between internal stress and deformation is not one-to-one, showing viscoelastic characteristics. Therefore, understanding the viscoelasticity during the fabrication process of fiber optic imaging arrays is crucial to avoid defects. However, studying the viscoelasticity of multi-component silicate glass is a challenging task due to the complex coupling of multiple components and factors, as well as the uncertainty between the elastic and viscous behaviors of the glass. Typically, researchers reduce interference by limiting the number of oxides introduced or setting fixed boundary conditions, thus enabling a better study of the relationship between composition and viscoelastic properties. Currently, the research in this area is still limited.

Previous studies have investigated the viscoelasticity of multi-component silicate glasses. For example, Eisenberg and Takahashi [10] explored the viscoelasticity of silicate glasses and found that the stress relaxation activation energy above the glass transition temperature was likely related to the Si-O bond energy. Kitamura [11] studied the viscoelasticity of Li^+^ and Na^+^ binary alkali silicate glasses, analyzing the effect of Li^+^ and Na^+^ contents on viscoelasticity using parallel-plate viscosity methods and dynamic viscoelastic measurements. Zhang et al. [12] proposed a minimum uniaxial creep test (MUCT) based on TMA, and validated the accuracy of the results using finite element simulations. Research on phosphate glasses [13], chalcogenide glasses [14], and borate glasses [15] has also provided valuable insights for the study of silicate glass viscoelasticity.

It can be seen that the existing research mainly focuses on the viscoelasticity of individual ions (such as Li^+^ and Na^+^), with limited studies on the effect of mixed alkali ions on viscoelasticity. The mixed alkali effect is an important physicochemical reaction involving the complex influence of multiple alkali metal ions on the structure and properties of glass. Previous studies have shown that the mixed alkali effect significantly impacts glass properties such as the coefficient of thermal expansion, glass transition temperature, and electrical conductivity [16].

In this study, Na⁺- and K⁺-doped 70 SiO_2_·(30 − x) Na_2_O·xK_2_O silicate glasses were prepared via the melting–quenching method to systematically investigate the mixed alkali effect on their viscoelastic behavior and structural evolution. The key parameters, including the glass transition temperature (*T*_g_) and dilatometric softening temperature (*T*_d_), were measured to design uniaxial compression creep experiments, which elucidated the impact of Na⁺/K⁺ substitution on viscoelasticity. In addition, Raman spectroscopy was employed to analyze the compositional effects on the glass network structure, establishing correlations between the composition and viscoelastic properties. These findings provide theoretical foundations and practical guidelines for optimizing the material design and fabrication processes of optical fiber imaging arrays.

## 2. Materials and Methods

### 2.1. Preparation of Silicate Glass

**Figure 1:**
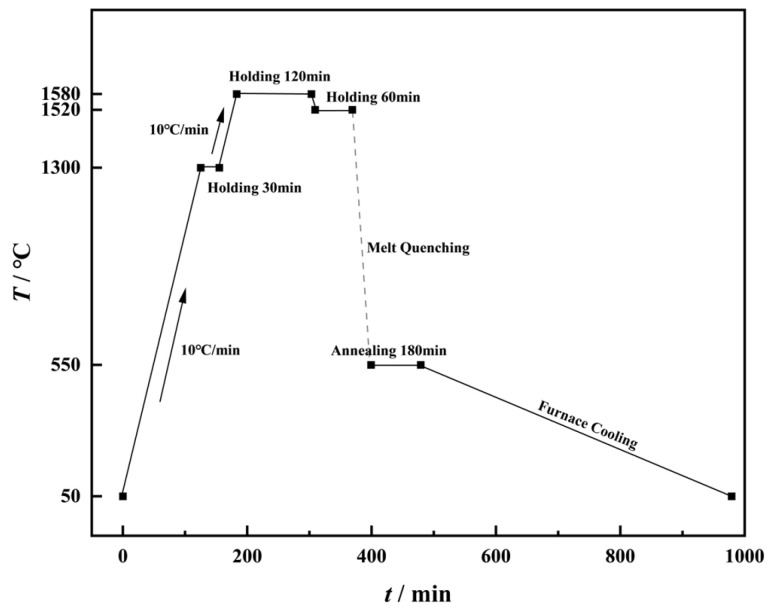
Schematic diagram of the glass making process.

A series of silicate glasses with the molar composition 70SiO_2_·(30 − x) Na_2_O·xK_2_O (where x = 0, 7.5, 15, 22.5, corresponding to the molar ratio *r* = [K_2_O]/([Na_2_O] + [K_2_O] = 0, 0.25, 0.5, 0.75) were prepared via the conventional melt–quenching method. Analytical purity grades of SiO_2_ (Sinopharm Chemical Reagent Co., Ltd., Shanghai, China)_._, Na_2_CO_3_ (Sinopharm Chemical Reagent Co., Ltd., Shanghai, China), and K_2_CO_3_ (Sinopharm Chemical Reagent Co., Ltd., Shanghai, China) were used as starting materials, with a total weight of 400 g. To ensure homogeneity, the weighed raw materials were placed in a zirconia ball-milling jar with zirconia grinding balls and mixed thoroughly for 5 h using an omnidirectional planetary ball mill (QXQM-2, Changsha Tianchuang Powder Technology Co., Ltd., Changsha, China). The homogenized mixture was transferred to a preheated platinum–rhodium alloy crucible at 1300 °C, held for 30 min, then heated to 1520 °C at 10 °C/min and maintained for 2 h to homogenize the melt and eliminate bubbles (as shown in Figure 1). Subsequently, the temperature was reduced to 1520 °C at 10 °C/min, held for 1 h, and the molten glass was cast into preheated graphite molds. The as-cast glasses were annealed at their respective glass transition temperatures for 3 h to relieve internal stresses, followed by furnace cooling. The annealed glasses were cut and polished to the required dimensions for further characterization.

### 2.2. Sample Characterization and Testing

The glass transition temperature (*T*_g_), dilatometric softening temperature (*T*_d_), and coefficient of thermal expansion (*CTE*, averaged over 30–300 °C) were measured using a thermal dilatometer (Netzsch DIL402 Classic, Selb, Germany) with a displacement resolution of 2 nm and a temperature accuracy of 0.1 K. The *CTE* was calculated using Equation (1):

(1) $$\alpha =\frac{1}{L_{0}}\cdot \frac{L-L_{0}}{T-T_{0}}$$

where α is the *CTE* (1/°C or 1/K), *L* and *L*_0_ are the sample lengths at the test and initial temperatures, and *T* and *T*_0_ are the corresponding temperatures. The measurements were conducted under a nitrogen atmosphere at a heating rate of 5 K/min using cylindrical samples (φ6 × 50 mm).

The softening point temperature (*T*_f_, corresponding to a viscosity of 10^6.6^ Pa·s) was determined using a high-temperature viscometer (Orton PPV-1000, Westerville, OH, USA) following ASTM C1351M. Cylindrical samples (φ6 × 6 mm) were compressed between parallel plates under controlled heating. The viscosity (*η*) was calculated using Equation (2) [17]:

(2) $$\eta =\frac{2\pi mgh^{5}}{3V\left(\frac{dh}{dt}\right)\left(2\pi h^{3}+V\right)}$$

where *m* is the applied load (kg), g is gravitational acceleration (m/s^2^), *t* is time (s), *h* is the instantaneous sample height (m), and *V* is the sample volume (m^3^).

Uniaxial compressive creep tests were performed using a thermomechanical analyzer (TMA, NETZSCH 402F1 Hyperion, Selb, Germany) with a displacement resolution of 0.125 nm and load resolution of 0.01 mN. Polished cylindrical samples (φ5 × 5 mm) were heated to target temperatures under nitrogen at 5 °C/min, equilibrated for 30 min, and subjected to a 2 N constant load for 90 min while recording their time-dependent deformation.

The nonlinear mechanical behavior of viscoelastic materials arises from their time-dependent stress–strain relationships, primarily manifested as two phenomena: creep (time-dependent strain accumulation under constant stress) and stress relaxation (time-dependent stress decay under constant strain). To quantify these behaviors, creep compliance *J*(t) = *ε*(t)/σ_0_ (strain response to constant stress *σ*_0_) and the shear relaxation modulus *G*(t) = *σ*(t)/*ε*_0_ (stress decay under constant strain *E*_0_) were introduced. To analyze the deformation accurately, true stress (σ_true_) and true strain (ε_true_) were calculated using Equations (3) and (4):

(3) $$\sigma_{true}=\sigma *\left(1+\varepsilon \right)=\frac{F}{A_{0}}*\left(1+\frac{△L}{L_{0}}\right)$$

(4) $$\varepsilon_{true}=\ln\left(1+\epsilon \right)=\ln\left(1+\frac{△L}{L_{0}}\right)$$

where *F* is the instantaneous load, *A*_0_ is the initial cross-sectional area, and *L*_0_ and ΔL are the initial height and deformation, respectively. Following compression creep tests, the creep compliance *J*(t) was obtained. Based on linear viscoelastic theory and referencing the methodologies of Arai and Zhang et al. [18,19], the creep curves *J*(*t*) were fitted using a four-element Kelvin model and calculated using Equation (5):

(5) $$J(t)=\frac{1}{E_{0}}-\sum_{i=1}^{4}\frac{1}{E_{i}}\left(1-\exp\left(-\frac{t}{\tau_{i}}\right)\right)$$

In this section, *E*_0_ represents the initial Young modulus, while *E_i_* and *τ_i_* characterize the elastic modulus and relaxation time constant of the *i*-th Kelvin unit, respectively. Finally, the shear relaxation modulus *G*(t) was derived via Laplace transform, where *G*(t) was defined as the stress relaxation under constant strain.

Raman spectra were acquired at room temperature using a Horiba HR Evolution spectrometer (Irvine, CA, USA) with a 532 nm semiconductor green laser (100 mW power) in the backscattering geometry. The spectra (200–1600 cm^−1^) were collected with a 1 cm^−1^ resolution, 15 s acquisition time, and were averaged over 200 scans to enhance the signal-to-noise ratio.

## 3. Results and Discussion

### 3.1. Viscoelastic Analysis

#### 3.1.1. Thermodynamic Analysis

**Figure 2:**
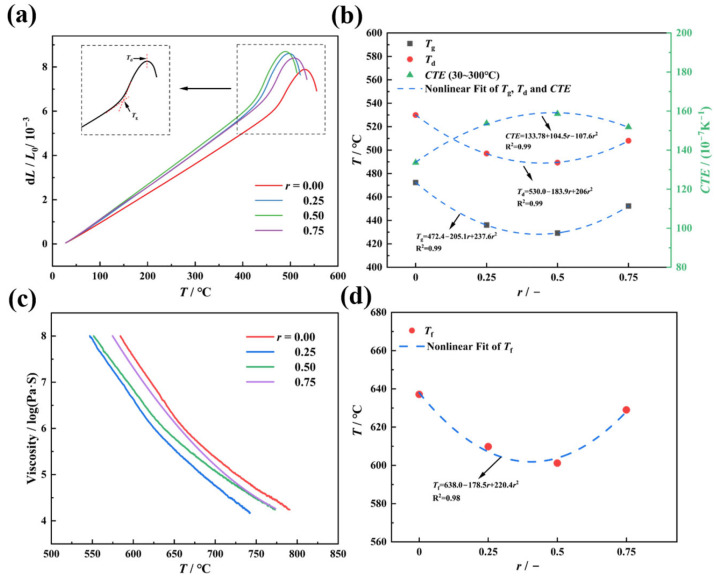
Thermal expansion and mid-temperature viscosity tests were conducted for 70SiO_2_ (30 − x) Na_2_O·xK_2_O glasses with varying molar ratios *r*, where *r* is equal to the molar ratio of [K_2_O]/([Na_2_O] + [K_2_O]). (**a**) Thermal expansion curves, with the inset illustrating the method of determining *T*_g_ and *T*_d_ from the expansion data; (**b**) variation in *T*_g_, *T*_d_, and the coefficient of thermal expansion *CTE* as a function of *r*; (**c**) mid-temperature viscosity curves; and (**d**) variation in *T*_f_ with *r*.

The state and mechanical properties of glass evolve with temperature. Below the glass transition temperature (*T*_g_), glass behaves predominantly as an elastic solid, while above the softening temperature (*T*_f_), it transitions to a viscous fluid. Between *T*_g_ and *T*_f_, glass exhibits complex viscoelastic behavior [20,21]. Thus, identifying its characteristic temperatures is critical for understanding its viscoelasticity. Figure 2a displays the original thermal expansion curve, with the inset illustrating the determination of *T*_g_ and the dilatometric softening temperature (*T*_d_). Figure 2b shows the composition dependence of *T*_g_, *T*_d_, and the coefficient of thermal expansion (*CTE*) as a function of *r* (the molar ratio of K_2_O/(Na_2_O K_2_O)). As *r* increases (i.e., K_2_O progressively replaces Na_2_O), *T*_g_ and *T*_d_ exhibit a “V”-shaped trend—initially decreasing, reaching minima at *r* = 0.5, then increasing. Conversely, *CTE* follows an inverse trend, peaking at *r* = 0.5 with values of 429.2 °C (*T*_g_), 489.3 °C (*T*_d_), and 158.63 × 10^−7^/°C (*CTE*), which is consistent with prior studies [22]. Similarly, *T*_f_, measured via parallel-plate viscometry (Figure 2c), shows a “V”-shaped dependence, reaching a minimum of 599.2 °C at *r* = 0.5 (Figure 2d). Notably, the composition-dependent trends of *T*_g_, *T*_d_, *CTE*, and *T*_f_ are well fitted by quadratic polynomial equations (*R*^2^ > 0.99), indicating a synergistic dual-variable regulation mechanism.

These phenomena can be attributed to dynamic structural changes in the glass network. At low *r* values, the partial substitution of Na^+^ by K^+^ disrupts the Na^+^-dominated network topology, increasing local structural strain and mismatch. This enhances network flexibility while introducing heterogeneous ion migration paths due to the “site memory effect” of mixed alkali ions. Specifically, alkali ions occupy sites with differing chemical environments and energy states, creating kinetic barriers for ion migration. Consequently, K^+^ incorporation disrupts uniform ion distribution, reducing *T*_g_, *T*_d_, and *T*_f_, while increasing *CTE* [23]. At *r* = 0.5, structural strain and mismatch are maximized, corresponding to the extremal values of *T*_g_ *T*_d_, *T*_f_, and *CTE*. As the K^+^ content further increases, it becomes the dominant network-modifying ion, gradually restoring structural homogeneity and reducing migration barriers. This leads to rising *T*_g_, *T*_d_, and *T*_f_, alongside declining *CTE* [24,25]. Within the *T*_g_–*T*_f_ range, the mechanical response of glass is governed by the interplay between network rearrangement and ion migration, resulting in intricate viscoelastic behavior. A detailed understanding of viscoelastic constitutive relationships in this regime is essential for optimizing the thermoforming processes and enhancing thermal stability.

#### 3.1.2. Creep and Stress Relaxation Analysis

**Figure 3:**
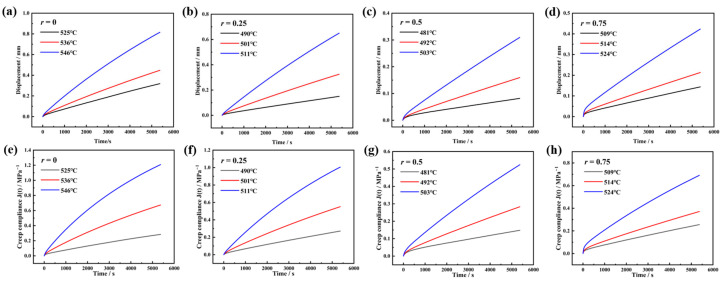
The history of height displacements and the calculated creep compliance curves of 70SiO_2_·(30 − x) Na_2_O·xK_2_O glass with different molar ratios r are measured, where *r* is equal to the molar ratio of [K_2_O]/([Na_2_O] + [K_2_O]). (**a**–**d**) Displacement history. (**e**–**h**) Creep compliance curves.

Figure 3a–d show the uniaxial compressive creep deformation curves of the glasses near their *T*_d_. As experimental temperature increases, the deformation magnitude under constant load grows, while the deformation rate decreases over time. The derived creep compliance *J*(t) is shown in Figure 3e–h. At lower experimental temperatures, *J*(t) exhibits linear growth, transitioning to nonlinear behavior at elevated temperatures.

**Figure 4:**
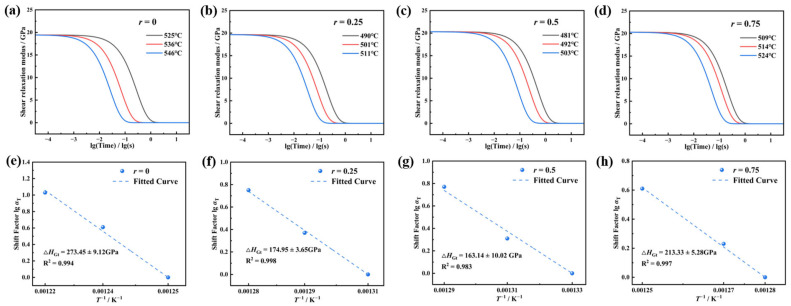
The relationship between the shear relaxation modulus *G*(t), the displacement factor lg α_T_, and the reciprocal of temperature *T* of 70SiO_2_·(30 − x) Na_2_O·xK_2_O glass with different molar ratios *r*, where *r* is equal to the molar ratio of [K_2_O]/([Na_2_O] + [K_2_O]). (**a**–**d**) Shear relaxation modulus *G*(t) curve. (**e**–**h**) Displacement factor lgα_T_ and the reciprocal of temperature T.

Figure 4a–d present the shear relaxation modulus *G*(t) calculated via Laplace transform for the glass systems near their dilatometric softening temperature *T*_d_. The *G*(t) curves exhibit a rapid decay to near-zero equilibrium values within extremely short time scales under constant stress at elevated temperatures. All *G*(t) curves at different temperatures can be superimposed by shifting along the logarithmic time axis, confirming that the system obeys the time–temperature superposition principle (TTSP) [26]. Specifically, the relaxation rate varies with temperature according to the TTSP. With an increasing creep test temperature, all *G*(t) curves shift toward shorter timescales, indicating accelerated relaxation rates. The distance of this shift is referred to as the shift factor lgα_T_, which obeys the Arrhenius-type Narayanaswamy equation [27], as shown in Equation (6):

(6) $$\mathrm{lg}\alpha_{T}\left(T\right)=\frac{{\Delta H}_{Gt}}{R}\left(\frac{1}{T_{0}}-\frac{1}{T}\right)$$

where *R* is the molar gas constant, 8.314 J·K^−1^·mol^−1^, and *T*_0_ is the initial temperature, which in this study refers to the glass’s dilatometric softening temperature *T*_d_, measured in Kelvin (K). Δ*H*_Gt_ represents the relaxation activation energy, which quantitatively characterizes the minimum energy required to overcome the barrier for network rearrangement.

Figure 4e–h show the relationship between the shift factor lg*α*_T_ and the reciprocal of temperature for *r* = 0, 0.25, 0.5, and 0.75, and the corresponding Δ*H*_Gt_ values are obtained by fitting with Equation (4). When *r* = 0, Δ*H*_Gt_ is approximately 273.45 kJ/mol, which is in close agreement with the dissociation energy of the Na-O bond (270 ± 4 kJ/mol [28]), indicating that the Na^+^ migration dominates the relaxation process in this composition. As K^+^ gradually replaces Na^+^, Δ*H*_Gt_ exhibits a trend of initially decreasing and then increasing, reaching a minimum of 163.14 kJ/mol at *r* = 0.5, which is significantly lower than in the single alkali system. When *r* = 0.75, it increases to 213.33 kJ/mol, which is slightly lower than the dissociation energy of the K-O bond (271.5 ± 12.6 kJ/mol [28]). This trend is highly consistent with the “V”-shaped variation in *T*_g_, *T*_d_, and *T*_f_, and also reveals that the mixed alkali effect significantly alters the network rearrangement barrier by modulating the ion migration synergy. Further analysis with chemical bond energies shows that the Si–O bond dissociation energy (798 kJ/mol [29]) is much higher than that of the R–O bonds (R = Na/K), and that the ionic bond characteristics of R–O bonds allow for greater bond length and bond angle tolerance. In contrast, the covalent nature of Si–O bonds make them more rigid. Therefore, at high temperatures, the viscoelastic relaxation process of glass is dominated by R–O bonds. By controlling the *r* value to optimize Δ*H*_Gt_, the gradient matching of the viscoelastic behavior of the core and clad glasses during hot forming can be optimized, providing a theoretical foundation for material design in the development of ultra-low-defect fiber optic imaging arrays.

### 3.2. Structural Origins of Viscoelastic Behavior

**Figure 5:**
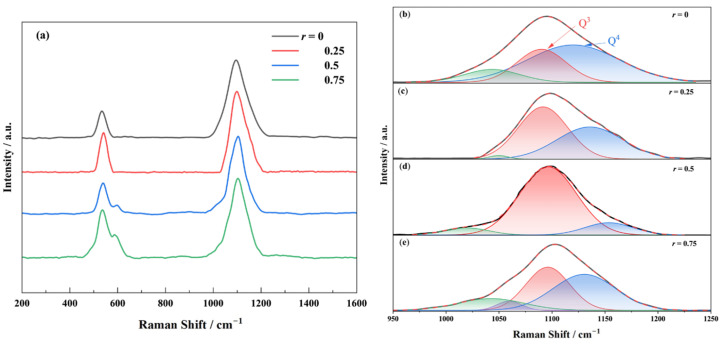
The Raman spectra of 70SiO_2_·(30 − x) Na_2_O·xK_2_O glass with different molar ratios *r* and the diagram of the results of Gaussian function fitting of the characteristic peak at 950–1250 cm^−1^ are obtained, where *r* is equal to the molar ratio of [K_2_O]/([Na_2_O] + [K_2_O]). (**a**) Raman spectra; and (**b**–**e**) *r* = 0, 0.25, 0.5, 0.75, respectively, in which the black line is the original Raman spectrum, the red region (1100 cm^−1^) corresponds to the Si-O stretching vibration in Q^3^ unit, the blue region (1150 cm^−1^) corresponds to the Si-O stretching vibration in Q^4^ unit, and the green region (1050 cm^−1^) is determined by the Si-O stretching vibration of Q^3^ and Q^4^.

**Figure 6:**
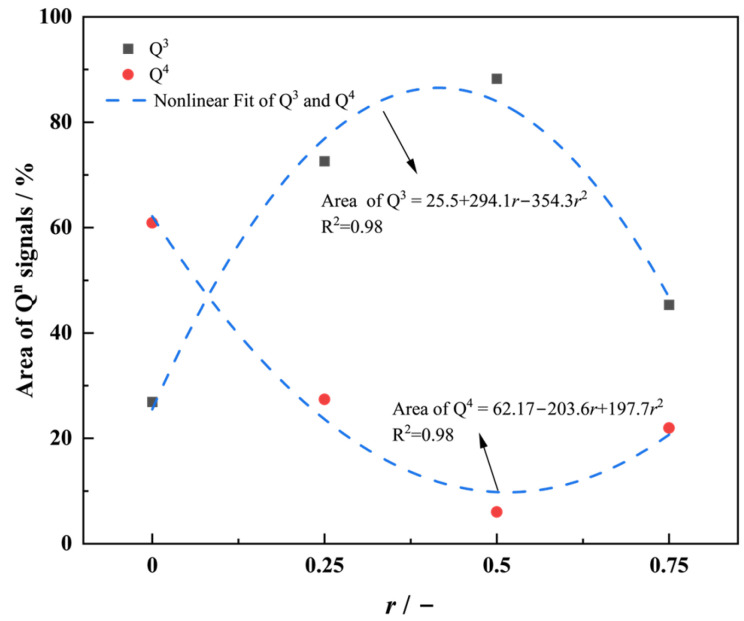
Q^3^ and Q^4^ unit area ratio versus *r* curve.

To elucidate the structural evolution induced by Na_2_O substitution with K_2_O, the Raman spectra of the 70SiO_2_·(30 − x) Na_2_O·xK_2_O glasses with varying *r* are analyzed (Figure 5a). Two dominant vibrational bands are observed: a low-frequency band at 400–600 cm⁻^1^ and a high-frequency band at 1000–1200 cm⁻^1^. The 400–600 cm⁻^1^ band corresponds to the symmetric stretching vibrations of Si–O bonds, which can be deconvoluted into two peaks at 520 cm⁻^1^ (D_1_, planar four-membered rings) and 606 cm⁻^1^ (D_2_, planar three-membered rings) [30,31,32]. Notably, the D_2_ peak intensity increases with the K⁺ content. The high-frequency band (1000–1200 cm⁻^1^) arises from asymmetric Si–O stretching vibrations and is highly sensitive to the distribution of [SiO_4_] structural units [22,33,34,35]. Based on the number of bridging oxygens (BOs) per [SiO_4_] unit, the structural units are classified as Q^1^–Q^4^, corresponding to peaks at −870 cm⁻^1^ (Q^1^), −950 cm⁻^1^ (Q^2^), −1100 cm⁻^1^ (Q^3^), and −1150 cm⁻^1^ (Q^4^). The 1050 cm⁻^1^ peak reflects the contributions from both Q^3^ and Q^4^. The wavenumbers of the Raman spectra corresponding to the glass structural groups are shown in Table 1. For the 70SiO_2_·(30 − x) Na_2_O·xK_2_O glasses, the network is dominated by Q^3^ and Q^4^ units. Figure 5b–e present the deconvolution results of the high-frequency band using Gaussian fitting, and Figure 6 summarizes the relative area ratios of these structural units as a function of *r*.

As *r* increases, the glass network undergoes significant restructuring. At a low K_2_O content, the incorporation of K⁺ (larger ionic radius, lower field strength) disrupts the network by destabilizing Si–O–Si bond angles, breaking BO bonds in Q^4^ units and converting them into Q^3^ units, thereby decreasing the degree of network polymerization. At *r* = 0.5, the synergistic perturbation from mixed alkali ions maximizes network depolymerization, with Q^3^ reaching its highest relative proportion (88%). By further increasing the K⁺ content, K⁺ will act as a dominant network modifier, occupying non-bridging oxygen (NBO) sites due to its lower field strength. This promotes BO reformation, increasing Q^4^ content and enhancing the degree of network polymerization, stability, and the mechanical strength of the network.

This structural reorganization mechanism explains the nonlinear variation in viscoelastic parameters (e.g., Δ*H*_Gt_). By precisely tuning *r*, the network topology of clad glass can be optimized to balance formability and dimensional stability. Near *r* = 0.5, the moderately depolymerized Q^3^-dominated network ensures sufficient rheological flow during thermoforming while maintaining structural integrity upon cooling, enabling the synergistic control of defects in fiber optic imaging arrays.

## 4. Conclusions

This study systematically investigates the effects of K_2_O substitution for Na_2_O on the viscoelastic behavior of silicate glasses by tuning the molar ratio *r* = [K_2_O]/([Na_2_O] + [K_2_O] (0, 0.25, 0.5, 0.75). The glass transition temperature (*T*_g_), dilatometric softening temperature (*T*_d_), and softening point temperature (*T*_f_) exhibit a distinct “V”-shaped dependence on *r*, reaching minima at *r* = 0.5: *T*_g_ = 429.2 °C, *T*_d_ = 489.3 °C, *T*_f_ = 599.2 °C. The coefficient of thermal expansion (*CTE*) follows an inverse trend, peaking at *r* = 0.5 (158.63 × 10⁻^7^/°C). This nonlinear evolution highlights the significant regulatory role of the mixed alkali effect on the thermodynamic and mechanical properties of silicate glasses. The trend of Δ*H*_Gt_ follows the “V”-shaped behavior observed for *T*_g_, *T*_d_, and *T*_f_; initially decreasing and then increasing with the increasing *r*. The Δ*H*_Gt_ values are 273.45, 174.95, 163.14, and 213.33 kJ/mol, reaching a minimum at *r* = 0.5. Additionally, when *r* = 0, Δ*H*_Gt_ is approximately 273.45 kJ/mol, consistent with the Na-O bond dissociation energy ((270 ± 4) kJ/mol). As *r* increases, Δ*H*_Gt_ falls below the bond dissociation energies of the Na-O and K-O bonds, indicating that the mixed alkali effect reduces the energy barrier for ion migration and structural rearrangement. Raman spectroscopy further confirms that Δ*H*_Gt_ is governed by alkali–oxygen (R-O) bond dynamics rather than the rigid Si-O network. These results provide critical insights into the tailoring of the viscoelastic behavior of multi-alkali silicate glasses through compositional design, enabling optimized thermoforming processes for high-performance fiber optic imaging arrays. In future work, we plan to integrate the prepared sodium–potassium mixed silicate glasses with existing core and absorber glasses to fabricate an optical fiber imaging array. This will allow us to investigate the influence of viscoelastic compatibility among glass components on the performance of the optical fiber imaging array, thereby further enhancing its imaging quality and improving the overall performance of night vision and detection devices.

## Figures and Tables

**Table 1:** The wavenumbers of the Raman spectra and the corresponding glass structural groups.

Wavenumber/cm^−1^	Corresponding Structural Group
520	Symmetric stretching vibration of Si-O in a planar four-membered ring (D_1_) [30,31,32]
606	Symmetric stretching vibration of Si-O in a planar three-membered ring (D_2_) [30,31,32]
1050	Asymmetric Si-O stretching vibrations in any Q^n^ unit [22,33,34,35]
1100	Si-O stretching in Q^3^ units [32,33,34,35]
1150	Si-O stretching in Q^4^ units [33,34,35]

## Data Availability

The original contributions presented in this study are included in the article. Further inquiries can be directed to the corresponding authors.
